# Regional Initiatives in Support of Surveillance in East Africa: The East Africa Integrated Disease Surveillance Network (EAIDSNet) Experience

**DOI:** 10.3402/ehtj.v6i0.19948

**Published:** 2013-01-25

**Authors:** Maurice Ope, Stanley Sonoiya, James Kariuki, Leonard E.G. Mboera, Ramana N.V. Gandham, Miriam Schneidman, Mwihaki Kimura

**Affiliations:** 1 East African Community Secretariat, Arusha, Tanzania; 2 Kenya Medical Research Institute, Nairobi, Kenya; 3 National Institute for Medical Research, Dar es Salaam, Tanzania; 4 World Bank, Washington, D.C., United States; 5 Rockefeller Foundation, Kenya Office

**Keywords:** EAIDSNet, One Health, regional surveillance network, East Africa, EAPHLNP, disease surveillance, field simulation exercise

## Abstract

The East African Integrated Disease Surveillance Network (EAIDSNet) was formed in response to a growing frequency of cross-border malaria outbreaks in the 1990s and a growing recognition that fragmented disease interventions, coupled with weak laboratory capacity, were making it difficult to respond in a timely manner to the outbreaks of malaria and other infectious diseases. The East Africa Community (EAC) partner states, with financial support from the Rockefeller Foundation, established EAIDSNet in 2000 to develop and strengthen the communication channels necessary for integrated cross-border disease surveillance and control efforts. The objective of this paper is to review the regional EAIDSNet initiative and highlight achievements and challenges in its implementation. Major accomplishments of EAIDSNet include influencing the establishment of a Department of Health within the EAC Secretariat to support a regional health agenda; successfully completing a regional field simulation exercise in pandemic influenza preparedness; and piloting a web-based portal for linking animal and human health disease surveillance. The strategic direction of EAIDSNet was shaped, in part, by lessons learned following a visit to the more established Mekong Basin Disease Surveillance (MBDS) regional network. Looking to the future, EAIDSNet is collaborating with the East, Central and Southern Africa Health Community (ECSA-HC), EAC partner states, and the World Health Organization to implement the World Bank-funded East Africa Public Health Laboratory Networking Project (EAPHLNP). The network has also begun lobbying East African countries for funding to support EAIDSNet activities.

## Introduction

The East African Community (EAC) partner states (Kenya, Uganda, Tanzania, Rwanda, Burundi) share a similar disease profile (1). Communicable diseases remain a major public health problem in the region, with HIV/AIDS, malaria, tuberculosis, respiratory infections, and diarrheal diseases continuing to cause high morbidity and mortality. For example, East African countries have suffered a huge burden of cholera over the past several years due largely to poor sanitation and inadequate supplies of safe water (2). Between 2002 and 2006, most Tanzanian regions reported cholera cases; nine regions reported more than 2,000 cases (3). In 2009, several Kenyan districts suffered cholera outbreaks, with 274 deaths and approximately 11,000 cases reported (4). The risk factors for some of these diseases, including influenza, are different in East Africa than in other parts of the world, with HIV-infected persons being more susceptible (5). East Africa is heavily affected by the HIV pandemic.

The communicable disease burden in the region is made especially challenging by the fact that some disease outbreaks, like the viral haemorrhagic fevers, cross geopolitical borders of the EAC partner states (6–8). For example, in 2007 an outbreak of Rift Valley fever was reported in Kenya and Tanzania, resulting in more than 1000 cases and 300 deaths (9). Other viral haemorrhagic fevers with the potential to spread across borders that have been detected in East Africa include Ebola in Uganda in 2000 (Gulu, Masindi and Mbarara districts), 2007–2008 (Bundibugyo district), and 2011 (Luwero district) (10–12). Additionally, in 2007 two different outbreaks of Marburg virus were reported in Kamwenge district of Uganda (13).

Wild Polio Virus (WPV) has also been reported in EAC with evidence of spread across borders. In 2006, two cases of WPV were reported in northeastern Kenya (Garissa district) that were due to an importation from Somalia (14). In 2009 another 18 cases of WPV were reported in Northern Kenya (Turkana district) that were genetically linked to a strain circulating in South Sudan. Uganda, which was declared polio free in the year 2006, having reported the last case in 1996, reported four cases of WPV in 2010 that were genetically linked to the strain previously reported in Northern Kenya. In 2011, another case of WPV that was genetically linked to a circulating strain in Uganda was reported in Western Kenya (Rongo District) (15).

Most countries in the region lack incentives and resources to invest in cross-border interventions; and border areas tend to be inhabited by especially vulnerable human populations, including migrant and rural populations. The challenge is compounded by inadequate mechanisms for a regional approach to the prevention and control of communicable diseases. Consequently, East Africa is experiencing a general lack of preparedness to deal with public health emergencies occurring across international boundaries. Interventions are fragmented. Representatives from the ministries of health and academic institutions in Kenya, Tanzania, and Uganda formed the East African Integrated Disease Surveillance Network (EAIDSNet) (http://www.eac.int/eaidsnet) to address these challenges.

The main objectives of the EAIDSNet are to: i) enhance and strengthen cross-country and cross-institutional collaboration through regional coordination of activities for the prevention and control of diseases and through a One Health approach, ii) promote exchange and dissemination of appropriate information on Integrated Disease Surveillance (IDS) and disease control activities as per the WHO integrated disease surveillance and response strategy, iii) harmonize IDS systems, iv) strengthen capacity for implementing IDS and control activities, and v) ensure continuous exchange of expertise and best practices for IDS and control. This paper describes the history of EAIDSNet, major achievements and challenges, and strategies for sustainability.

## History and Governance

A series of malaria outbreaks in East Africa in the 1990s led to the formation of EAIDSNet (16–18). At that time, there was no surveillance system in place for early detection of malaria outbreaks (19). In February 2000, the Tanzania National Institute for Medical Research (NIMR) brought together representatives of the Ministries of Health and the national health research and academic institutions of Kenya, Tanzania and Uganda to discuss the need for concerted efforts to ensure that correct epidemiological information is obtained and shared among the partner states and to achieve synergy in disease control efforts. Recognizing the need for a shared plan for the identification, monitoring and control of diseases in the region, EAIDSNet was founded in 2000 with financial assistance from the Rockefeller Foundation (RF) (20).

**Fig. 1:**
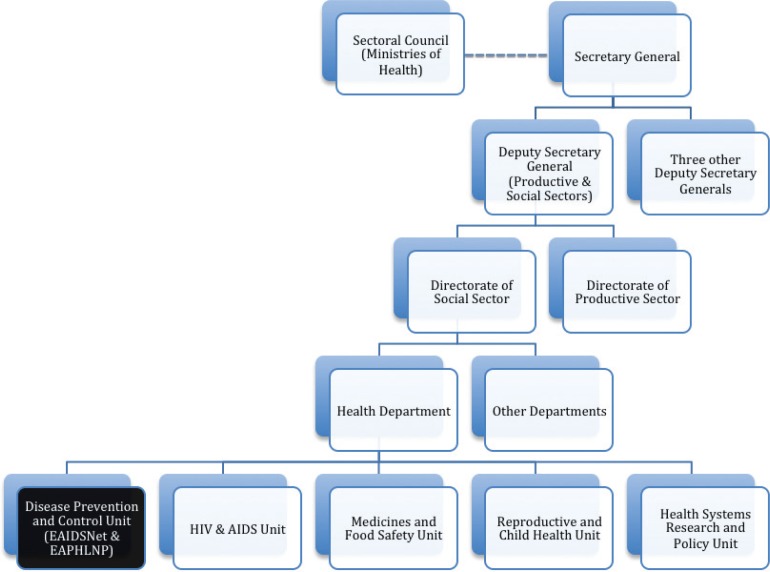
Organogram of the EAC indicating where EAIDSNet is situated. Source: EAIDSNet.

During the early days of the network, EAIDSNet activities were coordinated by the Tanzanian NIMR, which created a challenge for the sharing of information because not all member countries were obliged to submit surveillance reports to the Tanzanian NIMR. To address this, in 2003 EAIDSNet requested that a more regional body host the network. Subsequently, EAIDSNet was established under the auspices of the EAC, and coordination of activities was gradually moved from NIMR to EAC. In 2004, EAIDSNet was formally adopted by the EAC through the Council of Ministers, the second highest EAC organ after the Summit of EAC Heads of States. EAIDSNet activities became an integral part of the mandate of the disease prevention and control unit of the EAC health department (Figure 1; Text Box 1). Today, EAIDSNet activities must be approved by the EAC health sector coordinating committee, and all funds received by EAIDSNet must be included in the EAC budget and approved by the East African Legislative Assembly before activities are implemented.

***Text Box 1.* EAIDSNet led to the creation of a Health Department within the East African Community (EAC)**

The need to coordinate EAIDSNet activities at the regional level created the demand for a public health specialist at the EAC. Consequently, EAIDSNet influenced and fast-tracked creation of a health department at the EAC. At the time of recruitment, the health specialist held both the animal and human health portfolios. Since then, through funding from different sources, additional officers have been brought on board to handle the different portfolios. Collaboration between the human and animal sectors has remained good, with One Health activities dominating the regional agenda.

In addition to further developing its governance structure and mechanism, in 2003–2006 EAIDSNet received additional support from RF to: (i) strengthen communication and collaboration in disease surveillance; (ii) strengthen the capacity of collaborating institutions and border district health management teams for strategic and operational approaches to disease surveillance and control; and (iii) collaborate on the development of staff training programs for implementation of disease surveillance and control activities.

Since 2006, EAIDSNet has focused most of its efforts on strengthening EAC preparedness to respond to regional and global infectious disease threats. In 2008 EAC began implementing the “Regional Project to Strengthen Cross-Border Human and Animal Disease Prevention and Control in the East African Community Partner States” (21). The mandate of EAIDSNet has also expanded to Rwanda and Burundi, who formally joined the EAC in 2009.

## Major Activities, Achievements, and Lessons Learned

Since the inception of EAIDSNet, recognition of the shared risk of public health threats in border areas has driven ongoing discussion about how to strengthen cross-border disease surveillance and response. Most recently, in June 2011, in collaboration with EAC partner states and under the auspices of the East Africa Public Health Laboratory Networking Project, EAIDSNet convened a meeting of laboratory and disease surveillance and response experts from the region. Government officials and development partners also attended the meeting. The objective was to agree on a framework for implementation of cross-border disease surveillance and joint outbreak investigations, including community involvement. Participants discussed harmonization of surveillance data collection and reporting, analysis, and dissemination; and joint outbreak investigation and response. Representatives from partner states made presentations followed by group discussions, plenary sessions, and in-country consultations.

During the meeting, partner states agreed on a framework for cross-border surveillance and response, which included identification of priority diseases for either immediate, weekly, or monthly reporting to the EAC Secretariat (See Table 1). Meeting participants also designed a schedule for cross-border meetings of adjoining border districts and for informal sharing of disease outbreak alerts; and discussed preparation for joint outbreak investigations and responses to diseases or threats occurring in cross-border districts (e.g., infectious diseases that incubate in one country but occur in another, diseases involving contact between people in neighboring countries). The framework for cross-border surveillance and response was developed within the context of IDSR and IHR (2005); however, it calls for information sharing not only through the IHR focal points, but also between neighboring cross-border districts.

**Table 1:** List of priority diseases for EAIDSNet cross-border disease surveillance.

		Frequency of Reporting		
1	Acute haemorrhagic fevers	Immediate	Weekly	
2	Cholera	Immediate	Weekly	
3	Yellow fever	Immediate	Weekly	
4	Measles	Immediate	Weekly	
5	Plague	Immediate	Weekly	
6	(AFP) Poliomyelitis^1^	Immediate	Weekly	
7	Bloody diarhoea		Weekly	
8	Cerebro-spinal meningitis		Weekly	
9	Neonatal tetanus		Weekly	
10	Rabies (animal bites)		Weekly	
11	Malaria		Weekly	
12	Typhoid fever			Monthly
13	Diarrhea in <5 years			Monthly

In addition to achieving consensus on a framework for cross-border surveillance, the 2011 meeting resulted in the following recommendations for action, with some steps already taken:

- Seek endorsement of the framework for cross-border surveillance from member country Ministers of Health in order to enable formal sharing of information across borders. Following the meeting, partner states initiated in-country consultations to discuss the agreed upon framework and mobilize broad-based endorsement.
- Put together a regional rapid response team to conduct joint investigation of outbreaks in cross-border zones. Since the meeting, cross-border disease surveillance and response committees have been formed in the borders between Kenya and Tanzania, Kenya and Uganda, and Rwanda and Uganda. Similar rapid response teams will be formed between Tanzania and Rwanda, Tanzania and Burundi, and Rwanda and Burundi.
- Develop a regional mobile phone and web-based disease surveillance reporting system. A joint surveillance and information communication technology technical working group meeting was convened from April 30-May 3, 2012, during which technical user specifications were developed. A consultant will be hired to develop and deploy the regional mobile phone and web-based reporting system for surveillance data. The application used for the system will be enabled for both French and English languages. There will be further consultations with the countries, as the system is being developed to ensure it is acceptable to all stakeholders and implementation is on-track.

### Case Study 1: Experiences of a Field Simulation Exercise of the Kenyan and Ugandan National HPAI Preparedness and Response Plans

Here we describe the experience of a simulation exercise conducted in 2010 to test the Kenyan and Ugandan national highly pathogenic avian influenza (HPAI) preparedness and response plans. The simulation exercise demonstrated EAIDSNet's role in facilitating multi-country joint testing of both national and regional preparedness plans for pandemic influenza; and highlighted areas for improvement.

First detected in Hong Kong in 1997 (22), highly pathogenic avian influenza (HPAI) has been detected in over 22 countries. Approximately 566 cases and 332 deaths have been reported in 15 countries (23). In addition to its high case fatality rate (60 percent), HPAI has been associated with a high economic burden amounting to an estimated loss of USD 20 billion primarily due to the culling of several millions of birds. While international efforts have led to widespread control of HPAI, the disease persists in several countries, including Egypt and Indonesia, and continues to pose a threat to animal and human health. Although the EAC has not experienced any documented cases of HPAI, the region is vulnerable because of its location in the migratory pathway of birds, its shared borders with high-risk countries, and continued importation of poultry products that may carry the virus.

**Fig. 2:**
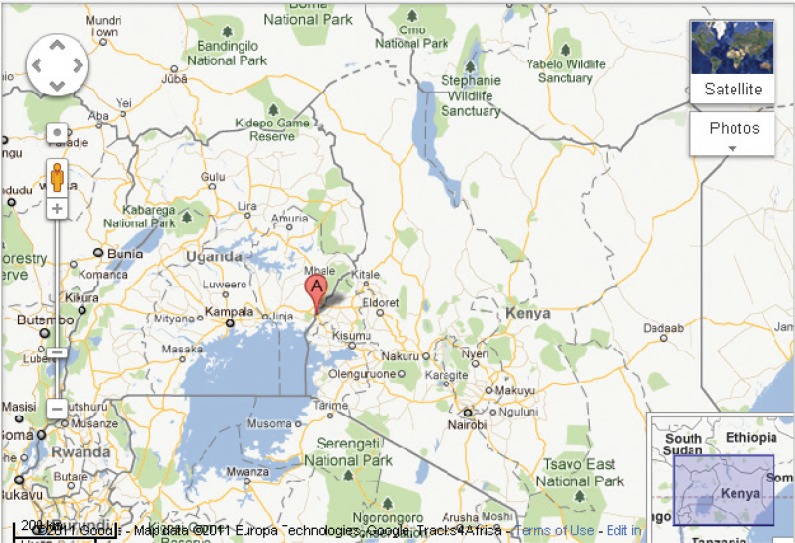
Map showing the Kenya-Uganda border town of Busia, where EAIDSNet conducted a field simulation exercise to test the Kenyan and Ugandan HPAI preparedness and response plans. Source: Google Maps.

EAIDSNet conducted one of the first field simulation exercises (FSXs) designed to test the effectiveness and efficiency of EAC partner state national HPAI preparedness and response plans. The focus of the exercise was on Kenya and Uganda. The FSX was conducted in Busia (Figure 2), a metropolitan border town between Kenya and Uganda. Busia lies within the migratory pathway of birds, has a thriving informal cross-border live bird market, and is home to many poultry farms. The exercise involved assessing the investigation and response of both countries to an imaginary scenario of a zoonotic public health emergency. Specific objectives of the FSX were to determine whether procedures were realistic and understood by all stakeholders; to reveal weaknesses and gaps; and to clarify roles and responsibilities of all key stakeholders.

The scenario for the simulation exercise was developed by experts from Food and Agriculture organization with the participation of EAIDSNet. It involved a report of bird mortality in a fish farm, followed a few days later by reports of significant mortality in a nearby backyard poultry farm and in a nearby commercial poultry farm. Meanwhile, the backyard poultry farmer had sold some of his chickens in a live bird market in Kenya. Subsequently there was a massive death of caged poultry in the bird market. Two traders from the market complained of fever, cough and sore throat and were treated at a private clinic. A few days later, the traders developed severe chest complications. The district veterinary officer was made aware of the two traders during his routine inspection of the market, after which he informed the clinician in charge of the local health center about the situation and referred the two traders to the health center. An evaluation criterion to determine the success of each operation was developed prior to the simulation exercise.

Several teams composed of staff of various disciplines, from both countries were formed to respond to the situation: veterinary, public health, communication, and security and biosecurity. Each team had specific roles and responsibilities to carry out. The veterinary team conducted investigations among both domestic and wild birds; identified and isolated infected areas; collected fecal, oral and blood samples from suspected birds; and confirmed HPAI at the central laboratory. They subsequently arranged for quarantine of birds at the live bird market, safe and timely disposal of carcasses, installation of footbath devices, provision of personal protective equipment, and disinfection of cages and affected areas.

The public health team conducted investigations and clinical assessments; transported suspected human cases to a designated health facility; set up an appropriate isolation unit and isolated patients; took samples for testing; and disinfected the ambulance. The communication team was responsible for producing and distributing paper and media communication; preparing and installing notice boards at crossing points; and creating public awareness through fliers, posters, and drama. Finally, the security and biosecurity teams were responsible for controlling traffic at the border and checking to see whether poultry products were being carried on board; closing some routes to the informal live bird market in order to enable thorough inspection of the vehicles; installing car footbaths; and disinfecting vehicles.

The FSX proved to be an effective method of testing regional preparedness and response. It demonstrated that control of border trade is possible in the event of an outbreak; that the synergistic roles of the different teams can be realized if the teams are composed of human and animal experts from both sides of the border; and that it is possible to increase public awareness of the risk of emergence and spread of HPAI and of the identification of areas where appropriate responses are required.

However, the exercise also revealed some weaknesses: overall poor coordination of the response activities, inadequate biosecurity measures, poor communication, and minimal involvement of medical workers in response to the HPAI outbreak. To address these weaknesses, EAIDSNet recommended that each district set up permanent multi-sectoral rapid response teams; communication materials be translated into local languages that can be understood by illiterate communities; and instructions for roadblock operations be included in the preparedness and response plans.

### Case Study 2: Piloting a Web-Based Portal for Linking Animal and Human Health Disease Surveillance

In Text Box 2, we describe the design and implementation of a web portal for the linking and sharing of animal and human disease surveillance data. The design and piloting of the web portal demonstrates EAIDSNet's capacity to facilitate joint collaboration in developing a regional mobile phone and web-based system for reporting surveillance data and thus reducing significantly the cost if each country were to develop its own separate system.

***Text Box 2.* EAIDSNet information communication technology (ICT) survey and web portal development**

An important objective of EAIDSNet is to improve the flow and quality of data and the sharing of information on communicable diseases. To help achieve this objective, first we conducted an information communication technology (ICT) situation analysis among participating EAIDSNet institutions and determined level of ICT usage among health care providers. The survey revealed that computer literacy among health workers was high, but that reporting of surveillance data was paper-based, except in Zanzibar and Rwanda where an electronic system was in use. Other EAC countries were at various stages of implementing mostly open software electronic reporting systems. Then, based on results of the ICT analysis, we developed and piloted a web data portal linking existing human and animal disease surveillance reporting systems across health facilities in cross-border districts.

In preparation for development of the web portal, we held consultative meetings with healthcare managers, inter-government agencies, ICT solution providers, and telecommunication operators. We designed the web portal using Hypertext Preprocessor (PHP) web-authoring software (Adobe™ Dreamweaver™ CS3) and two types of mapping software (ArcView GIS-9™ and HealthMapper™). As part of the pilot phase, personnel from the Ministries of Health of the partner states captured public health priority disease data into the web portal.

The pilot EAIDSNet web portal successfully mapped trends over time for diseases in selected sub-regions, and cross-border health personnel were able to view epidemiological maps in real-time. However, we faced several challenges. These included country-specific reporting requirements; late submission of weekly epidemiological data from field stations to central units; a lengthy data validation process; and different agencies requiring different data. Together, the challenges created a “burn-out” effect on data personnel at the national level. A more user-friendly portal that could auto-populate data from existing country-specific and regional systems will be essential to ensuring sustained use of the EAIDSNet web portal.

## Relationship to Connecting Organizations for Regional Disease Surveillance (CORDS)

Through Connecting Organizations for Regional Disease Surveillance (CORDS) (24), knowledge sharing between EAIDSNet and older and more experienced regional disease surveillance networks, like the Mekong Basin Disease Surveillance (MBDS) network, helped shape the strategic direction of EAIDSNet and, over time, has enabled EAIDSNet to improve on other networks’ best practices. For example, CORDS and Rockefeller Foundation facilitated exchange visits between the EAIDSNet and MBDS networks; attendance by both networks at the Prince Mahidol Award Conference in 2010 and the East African Health and Scientific Conferences in 2009 and 2010; and joint desktop exercises. Similarly, younger disease surveillance networks, such as the nascent West Africa Disease Surveillance Network, have much to learn from EAIDSNet.

## Moving Forward

While EAIDSNet has accomplished several major undertakings in its early years, a major challenge still facing the network is that meetings are often attended by new delegates from the partner states, requiring that issues agreed upon in previous meetings be re-visited and thereby slowing implementation of regional activities. Also, institutional participation in EAIDSNet declined when EAIDSNet came under the auspices of EAC and when partner states’ Ministries of Health started determining who attends the meetings. This particularly affected the academic institutions whose operations are regulated by Ministries of Education or Higher Education and not Ministries of Health. Together, these challenges make it difficult to implement joint outbreak investigations of cross-border events.

Additionally, insufficient laboratory capacity remains a major weakness in regional surveillance of communicable diseases across the EAC. Much of the equipment is outdated or has not been serviced; and laboratory providers have little opportunity for career advancement. Upon realization of this weak link, the EAC, through EAIDSNet, has partnered with the East Central and Southern Africa Health Community (ECSA-HC) to create the World Bank-funded East African Public Health Laboratory Networking (EAPHLN) Project. By strengthening laboratory capacity, the aim of EAPHLN is to improve regional surveillance in East Africa. By doing so, EAPHLN will help to realize the vision of EAIDSNet. Also looking to the future, EAIDSNet has begun lobbying East Africa countries for funding to support EAIDSNet activities.
